# A Bowman–Birk inhibitor induces apoptosis in human breast adenocarcinoma through mitochondrial impairment and oxidative damage following proteasome 20S inhibition

**DOI:** 10.1038/cddiscovery.2015.67

**Published:** 2016-03-21

**Authors:** A Mehdad, Giselle Xavier Reis, AA Souza, JARG Barbosa, MM Ventura, SM de Freitas

**Affiliations:** 1 Laboratory of Molecular Biophysics, Institute of Biological Sciences, University of Brasilia, Brasilia, Brazil; 2 Faculty of Medicine, Department of Molecular Pathology, University of Brasilia, Brasilia, Brazil

## Abstract

Proteasome inhibitors are emerging as a new class of chemopreventive agents and have gained huge importance as potential pharmacological tools in breast cancer treatment. Improved understanding of the role played by proteases and their specific inhibitors in humans offers novel and challenging opportunities for preventive and therapeutic intervention. In this study, we demonstrated that the Bowman–Birk protease inhibitor from *Vigna unguiculata* seeds, named black-eyed pea trypsin/chymotrypsin Inhibitor (BTCI), potently suppresses human breast adenocarcinoma cell viability by inhibiting the activity of proteasome 20S. BTCI induced a negative growth effect against a panel of breast cancer cells, with a concomitant cytostatic effect at the G2/M phase of the cell cycle and an increase in apoptosis, as observed by an augmented number of cells at the sub-G1 phase and annexin V-fluorescin isothiocyanate (FITC)/propidium iodide (PI) staining. In contrast, BTCI exhibited no cytotoxic effect on normal mammary epithelial cells. Moreover, the increased levels of intracellular reactive oxygen species (ROS) and changes in the mitochondrial membrane potential in cells treated with BTCI indicated mitochondrial damage as a crucial cellular event responsible for the apoptotic process. The higher activity of caspase in tumoral cells treated with BTCI in comparison with untreated cells suggests that BTCI induces apoptosis in a caspase-dependent manner. BTCI affected NF-kB target gene expression in both non invasive and invasive breast cancer cell lines, with the effect highly pronounced in the invasive cells. An increased expression of interleukin-8 (IL-8) in both cell lines was also observed. Taken together, these results suggest that BTCI promotes apoptosis through ROS-induced mitochondrial damage following proteasome inhibition. These findings highlight the pharmacological potential and benefit of BTCI in breast cancer treatment.

## Introduction

Breast cancer represents the most common cancer among women worldwide, with an estimated 1.67 million new cases diagnosed in 2012.^1^ A growing body of evidence suggests that Bowman–Birk inhibitors (BBIs), a major protease inhibitor family, can prevent or suppress carcinogenic processes that include colon,^2–4^ oral leukoplakia,^5–9^ esophageal tumors,^10^ leukemia,^11^ prostatic hyperplasia^12^ and breast cancer.^13^ However, despite acting on wide range of cancers, the underlying mechanism(s) of BBI activities as an anti-carcinogenic agent remain elusive. Nevertheless, the reliable explanation today is that BBIs are effective inhibitors of proteasomal chymotrypsin-like activities.^14^

The proteasome is a multi-subunit protease with three catalytic sites located in different subunits of the 20S core. These comprise the caspase-like, trypsin-like and chymotrypsin-like, *β*1, *β*2 and *β*5 subunits, respectively.^15^ The ubiquitin-proteasome proteolytic pathway is the principal mechanism in the cell for controlled protein degradation. As such, it controls the proteins that are crucial in cellular processes such as cell cycle progression, cell growth and cell development. It is therefore expected that dysfunction of these pathways will compromise cell survival. Indeed, it is well known that proteasome inhibition induces apoptosis.^16–20^ Moreover, high-proteasome activity has been reported in different malignancies.^14^ Because cancer cells are more sensitive than normal cells to the inhibition of proteasome activity,^21,22^ targeting the proteasome pathway has emerged as an ideal approach in anticancer therapy.

It has been shown that proteasome dysfunction causes damage to mitochondria, leading to increase in reactive oxygen species (ROS) production.^23^ Mitochondria have a major role in regulating cell death, which is mediated by outer membrane permeabilization in response to death triggers such as DNA damage.^24^ ROS are natural compounds which occur as chemical derivatives of metabolism. Performing key roles in apoptosis induction under both physiologic and pathologic conditions;^25^ elevated levels of ROS and downregulation of ROS scavengers lead to oxidative stress, which is associated with several human diseases, including cancers.^26^ It has been demonstrated that protease inhibitors promote cell cycle arrest and apoptosis.^17,27–30^ Moreover, proteasome impairment is associated with mitochondrial dysfunction, oxidative stress and death in neuronal cells.^31,32^ Therefore, it seems plausible to assume that proteasome inhibition induces apoptosis through mitochondrial dysfunction and oxidative stress. Recently, it has been shown that oxidative stress can convert estrogen-dependent non-aggressive breast cancer cells into an estrogen-independent aggressive form.^33^ Furthermore, mitochondrial ROS have been implicated in malignant cell transformation.^34^ Mitochondrial dysfunction was also shown to promote breast cancer cell migration and invasion through the accumulation of the transcription factor hypoxia-inducible factor 1*α*, via increased production of ROS.^35^ Nevertheless, the exact mechanisms controlling ROS involvement in carcinogenesis remain unclear.

The black-eyed pea trypsin/chymotrypsin inhibitor (BTCI) is a protease inhibitor isolated from *Vigna unguiculata* (Cowpea) seeds that belongs to the BBI family. BTCI is a stable double-headed Bowman–Birk protease inhibitor, inhibiting trypsin and chymotrypsin, simultaneously, with low-molecular mass (9071 Da) and seven disulfide bonds. The high-disulfide bond content of BTCI is responsible for its remarkable stability and also for the canonic conformation of loops containing the reactive sites of the inhibitor against proteases.^36–38^ The biochemical and biophysical properties of BTCI have been extensively characterized.^36,39^ Previously, we have reported that BTCI-induced significant cytostatic and cytotoxic effects on MCF-7 breast cancer cells; with these effects associated with changes in the morphology of the nucleus and mitochondria, increased number of cells with reduced mitochondrial membrane potential, DNA fragmentation and cells with altered plasma membrane integrity.^13^ We have also observed that during chemical induction of non-melanoma skin cancer in mice, topical application of BTCI significantly reduced the incidence and the volume of pre-malignant lesions (data to be published). In another study, we have shown that BTCI potentially inhibits the activity of trypsin-like, chymotrypsin-like and caspase-like sites of proteasome 20S, suggesting that BTCI is an effective proteasome inhibitor.^40^

To gain further insights into pleiotropic effects of BTCI, in the present study, we demonstrated that BTCI induced G2 phase/mitotic arrest and apoptotic cell breast cancer death. Furthermore, BTCI treatment caused mitochondrial membrane depolarization, triggering oxidative stress and increased caspase-3 activity. NF-kB target gene expression was also altered in both breast cancer cell lines and increased gene expression observed in interleukin-8 (IL-8) cancer cells. These finding suggest that BTCI induces apoptosis through mitochondrial impairment and oxidative damage following proteasome inhibition. Altogether, the results support the idea that the chemopreventive effect of BBIs may be attributable to their inhibitory effects toward proteasome function.

## Results

### Purification of BTCI

**Figure 1:**
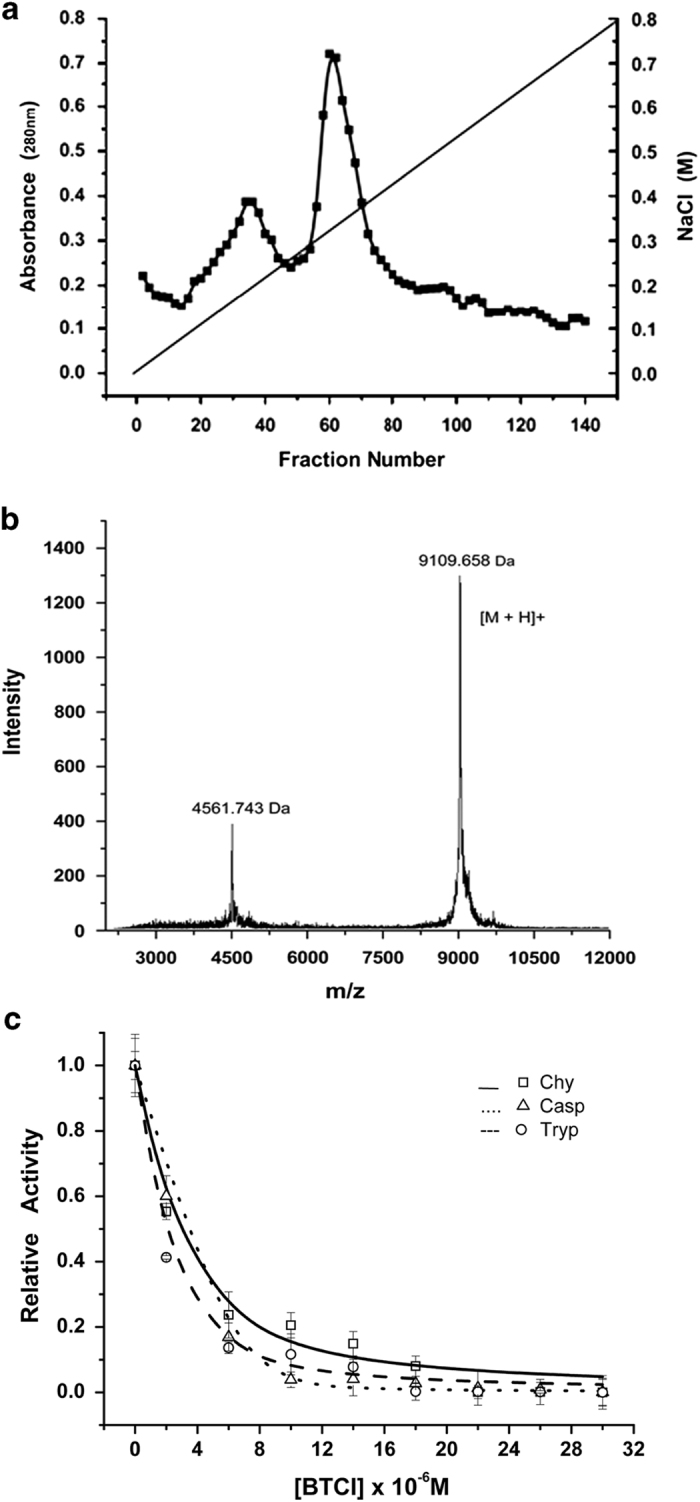
Purification and characterization of inhibitory activity of BTCI against proteasome 20S. (**a**) Elution protein profile from *V. unguiculata* seeds CE using DEAE-cellulose chromatography in accordance with Ventura and Xavier Filho (1996).^39^ BTCI was obtained from 67 to 87 eluted fractions. (**b**) MALDI-TOF MS spectrum showing BTCI as a pure protein of 9109.658 Da and its double charge [M + 2H] of 4561.743 Da.^38^ (**c**) Inhibition activity of trypsin-like, chymotrypsin-like and caspase-like sites by BTCI as previously described.^40^

The purification of BTCI from *V. unguiculata* seeds and the analysis of its purity were performed before testing in cell assays. The DEAE-cellulose elution profile of *V. unguiculata* seeds crude extract (CE), as previously described,^39,41^ is shown in Figure 1a. BTCI was collected between fractions 67 and 87 and its molecular mass (9071.6 Da) and purity were confirmed by MALDI-TOFF spectrometry (Figure 1b).^38^

### Inhibition of proteasome 20S function by BTCI

BTCI was previously characterized as a potent inhibitor of the 20S proteasome through the inhibition of protease trypsin, chymotrypsin and caspase-like activities,^40^ as shown in Figure 1c. BTCI showed high affinity to the 20S proteasome, as indicated by inhibition constant values of 1.0×10^−7^ M, 7.0×10^−7^ M and 14.0×10-^7^ M, for trypsin-like, chymotrypsin-like and caspase-like, respectively. It is noteworthy that BTCI was a more potent inhibitor for trypsin than known proteasome inhibitor N-(benzyloxy—carbonyl) leucinyl-leucinyl-leucinal-Leu-Leu-Leu-al (MG132), with similar inhibition observed toward chymotrypsin and caspase.

### BTCI-induced cytotoxicity in breast adenocarcinoma cells

**Figure 2:**
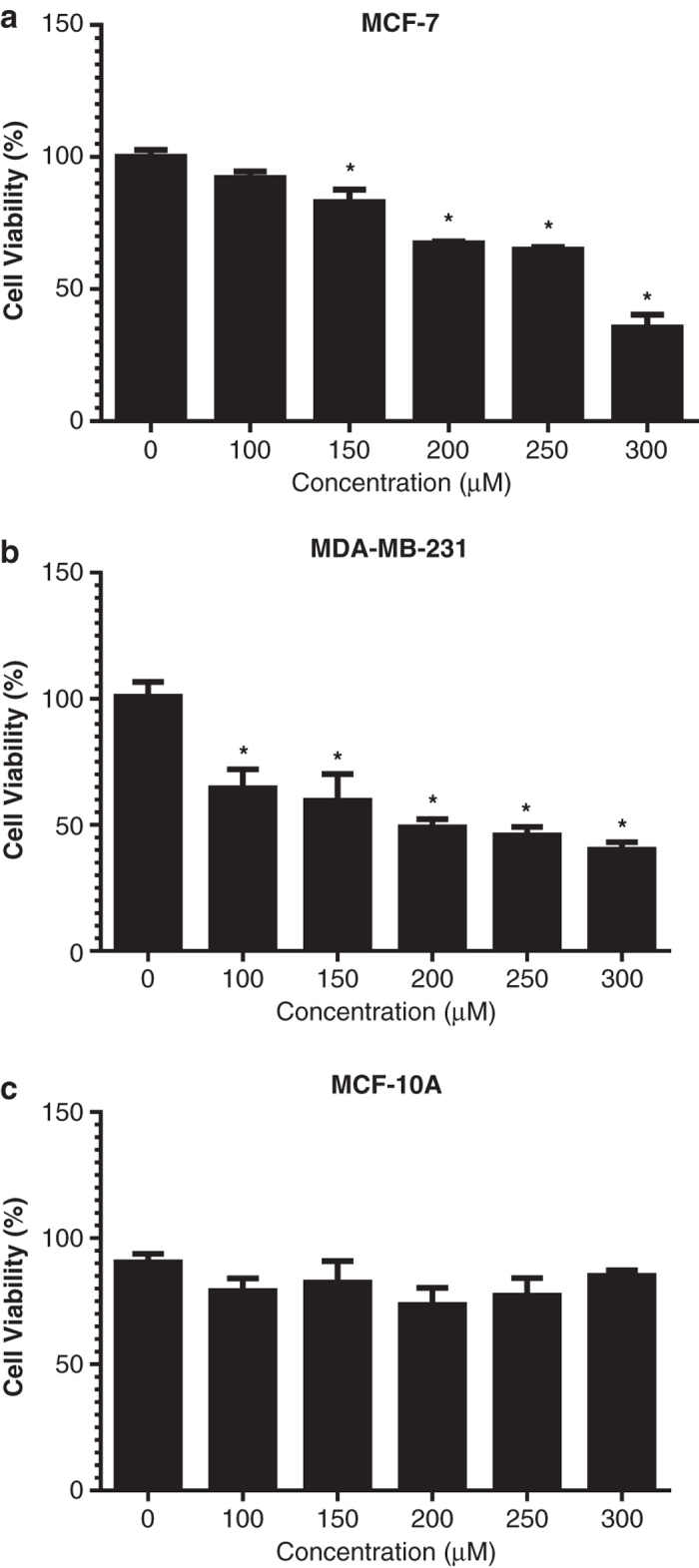
Cytotoxic effects induced by BTCI. (**a**) MCF-7, (**b**) MDA-MB-231 and (**c**) MCF-10 cells were incubated with BTCI (0-300 *μ*M) for 24 h. Cell viability was determined by MTT assay. Results are presented as mean±S.D. of two separate experiments conducted in triplicate, *P<0.05 *versus* untreated cells.

The effect of BTCI on viability of MDA.MB.231 (highly invasive human breast cells), MCF-7 (human breast adenocarcinoma cells) and MCF-10A (normal mammary epithelial cells) were determined using the MTT assay as described in the Methods section. Cells were incubated with BTCI at concentrations ranging from 0 to 300 *μ*M for 24 h. Results showed that BTCI exerts its cytotoxic effect in a dose-dependent manner. BTCI at the concentration of 150 *μ*M markedly induced MCF-7 death (*P*=0.014) (Figure 2a), meanwhile, the same effect on MDA.MB.231 was observed at a concentration of 100 *μ*M (*P*<0.001; Figure 2b). In contrast, BTCI did not induce a cytotoxic effect on MCF-10A cells. The results of the MTT assay demonstrated a cytotoxic effect of BTCI on breast cancer cell lines in a dose-dependent manner, absent in normal mammary epithelial cells (Figure 2c).

### BTCI-induced cell cycle arrest at G2/M phase and apoptotic cell death

**Figure 3:**
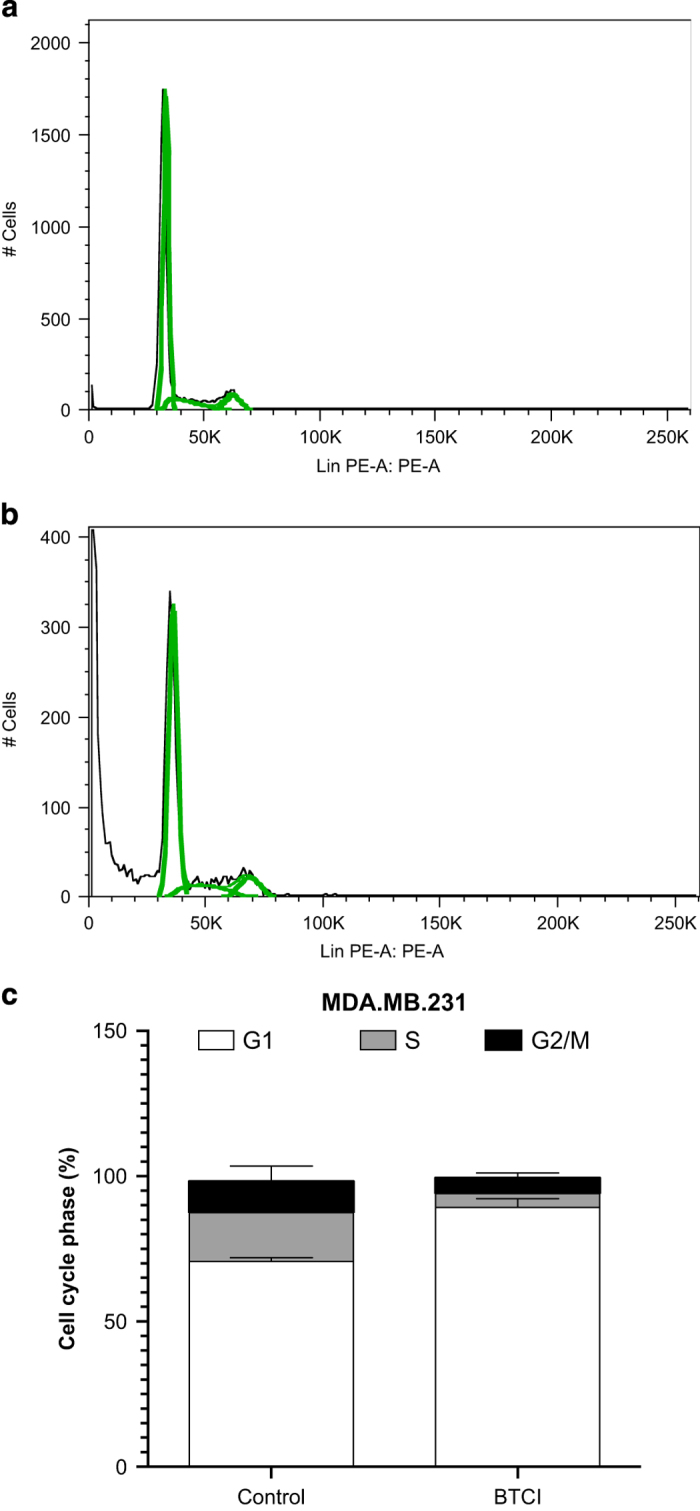
Effects of BTCI on DNA content. MDA-MB-231 cells were incubated with 100 *μ*M of BTCI for 24 h. DNA contents in Sub-G1 phase were analyzed using flow cytometry. Representative flow cytometry images were shown (**a** and **b**). (**c**) The DNA profile representing cells in sub-G1/G1, S and G2/M phases. Results are represented as means±S.D. of two separate experiments conducted in triplicate, *P*<0.05 *versus* untreated cells.

**Figure 4:**
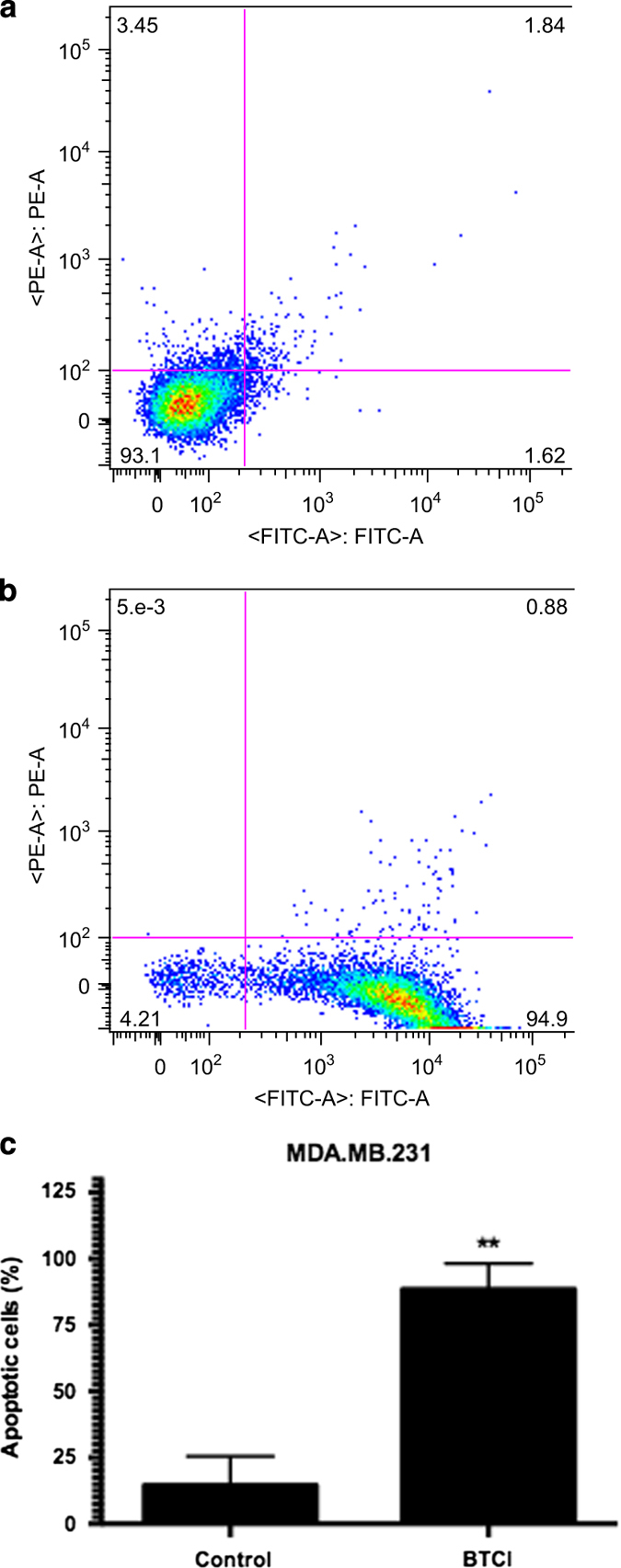
Apoptosis induced by BTCI MDA-MB-231. Cells were incubated with BTCI (100 *μ*M) for 24 h, and then stained with annexin V-FITC/PI and flow cytometry analysis. (**a** and **b**) representative images of flow cytometry analysis were displayed. (**c**) The percentage of apoptotic cells was statistically analyzed. Results are presented as mean±S.D. of two separate experiments conducted in triplicate, *P<0.001 *versus* untreated cells.

We previously showed that BTCI significantly induces cell cycle arrest at G2/M phase, DNA fragmentation and enhanced numbers of cells marked by annexin-V in breast cancer MCF-7, suggesting apoptosis as a possible mechanism for the cell death process.^13^ To explore whether apoptosis is involved in BTCI-induced MDA.MB.231, the amount of sub-G1 DNA in cancer cells treated with BTCI was investigated. As shown in Figure 3, the treatment of MDA.MB.231 cells with BTCI resulted in a marked accumulation of cells in the sub-G1 phase, suggesting a massive apoptotic, but not necrotic, cell death. In addition, BTCI arrested cell cycle at G2/M phase after 24 h. The apoptotic effect of BTCI was confirmed by an annexin V-fluorescin isothiocyanate (FITC)/propidium iodide (PI) staining assay. BTCI at the concentration of 100 *μ*M for 24 h induced apoptosis in MDA-MB-231 cells (Figure 4). Taken together, these results confirm that BTCI-induced apoptotic cell death in breast adenocarcinoma cells.

### Mitochondrial membrane depolarization is involved in BTCI-induced cytotoxicity

**Figure 5:**
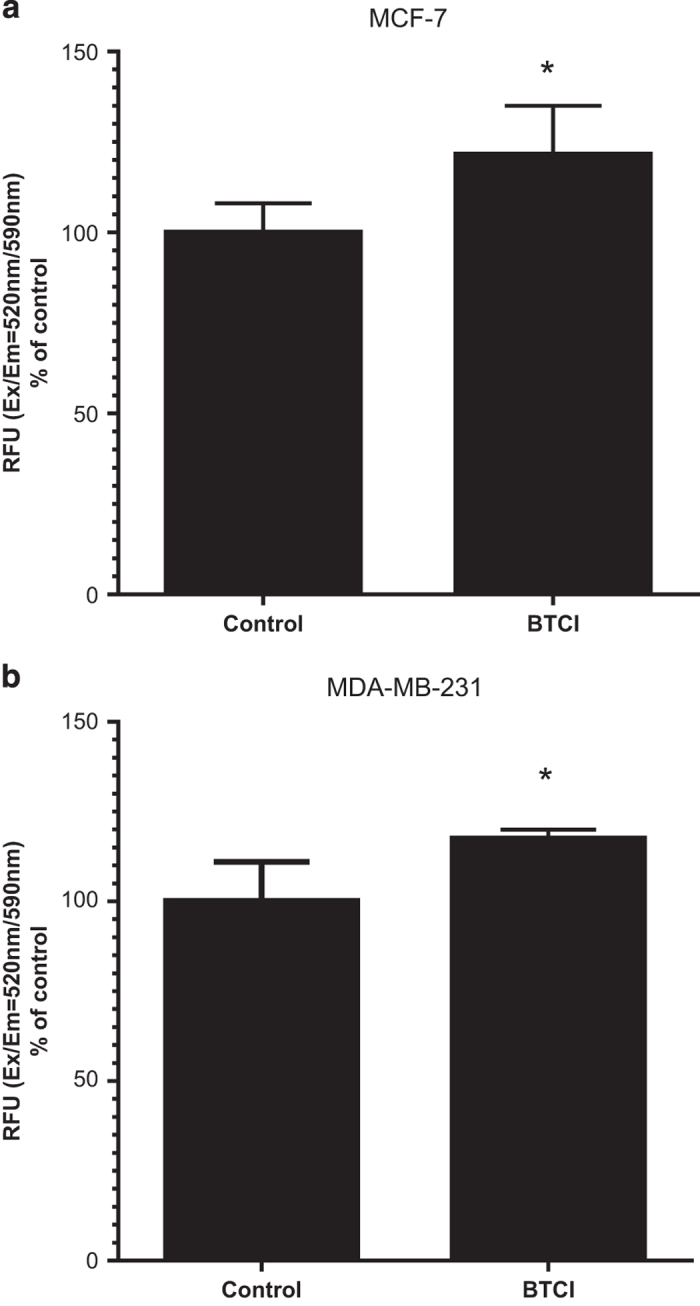
BTCI-induced mitochondrial membrane depolarization. (**a**) MCF-7 and (**b**) MDA-MB-231 cells were incubated with BTCI at the concentration of 150 *μ*M and 100 *μ*M, respectively, for 24 h. Dye-loading solutions were added to cells and incubated for 30 min. The change of mitochondrial membrane potential was measured by using Mitochondrial Membrane Potential Kit (Sigma). Results are presented as mean±S.D. of two separate experiments conducted in triplicate, *P<0.01 *versus* untreated cells.

Proteasome inhibition is known to induce apoptosis through mitochondrial cytochrome *c* release. As BTCI inhibits the proteasome function, its involvement in mitochondrial dysfunction in cancer cells was investigated. A sensitive cationic and lipophilic JC-10 fluorescent probe was used to monitor the mitochondrial membrane potential change in cells based on the presence of the JC-10 dye. JC-10 concentrates in the mitochondrial matrix where it forms red fluorescent aggregates. However, in apoptotic and necrotic cells, JC-10 diffuses out of mitochondria and changes from the aggregate to a monomeric form, causing a shift in fluorescence emission from ~590 to 515 nm, staining cells in green fluorescence. As shown in Figure 5, the mitochondrial membrane potential in both MCF-7 and MDA.MB.231 cells dropped, as indicated by increasing in monomer/aggregate ratio (*P*=0.016 and *P*=0.009, respectively). These data indicate that mitochondrial membrane depolarization and mitochondrial swelling precede an increase in oxidative stress and loss of cell viability in cells exposed to BTCI.

### BTCI increases intracellular ROS generation in breast adenocarcinoma

There is increasing evidence that ROS induced by apoptotic stimuli leads to mitochondrial dysfunction. To investigate the involvement of oxidative stress in BTCI-induced mitochondrial impairment as well as by proteasome inhibition, intracellular ROS levels were measured. Cells were treated with BTCI for 30, 60, 90, 120 and 180 min. As shown in Figure 5, treatment of breast adenocarcinoma cells with BTCI significantly increased intracellular ROS generation in a cell type and time point dependent manner. It is noteworthy that these results are strongly related with proteasome dysfunction-induced ROS and mitochondrial membrane depolarization in human breast adenocarcinoma.6

### BTCI treatment led to increased caspase-3 activity

**Figure 7:**
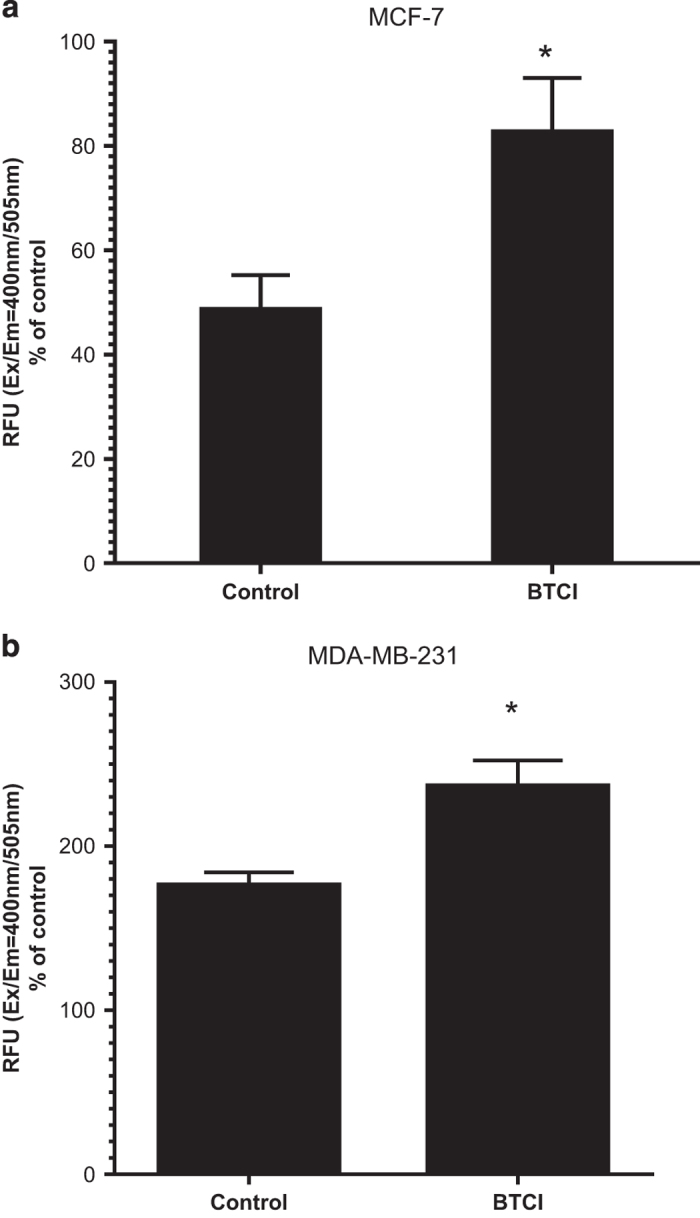
Effect of BTCI on caspase-3 activity. (**a**) MCF-7 and (**b**) MDA-MB-231 cells were incubated with BTCI at the concentration of 150 *μ*M and 100 *μ*M, respectively for 24 h; the activity of caspase-3 was evaluated by using caspase-3 activity kit. Results are presented as mean±S.D. of two separate experiments conducted in triplicate, *P<0.001 *versus* untreated cells.

To analyze whether BTCI triggers apoptosis in a caspase-dependent manner, the activity of caspase-3 was measured. As shown in Figure 7, the activity of caspase-3 in MCF-7 and MDA.MB.231 cells was increased after treatment with BTCI. Although MCF-7 does not express caspase-3, the increased activity observed in MCF-7 may be due to the assessment method via DEVD-dependent caspase activity. The assay is based on detection of the cleaved substrate DEVD-AFC (AFC: 7-amino-4-trifluoromethyl coumarin) that also can detect caspase-7 activity, which is expressed in MCF-7 cells and exhibits a certain degree of functional redundancy with caspase-3.

### Treatment with BTCI results in alteration on NF-kB dependent gene expression

**Figure 8:**
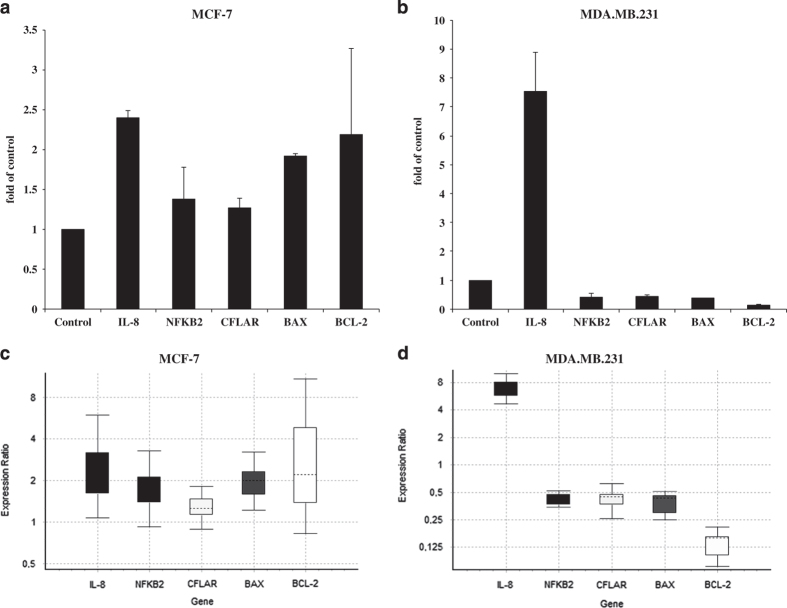
mRNA expression of IL-8, NFKB2, CFLAR, BAX and BCL-2 quantified by RT-PCR in human breast adenocarcinoma cells. Cells were treated with BTCI for 24 h, and RNA was extracted. This RNA was used to prepare cDNA that was subject to real-time PCR to quantify the mRNA level of each gene. Each sample was run in triplicate, and the relative amount of mRNA was normalized to the *β*-actin content in each sample. Normalized expression values (specific gene expression levels/*β*-actin expression levels) are presented. Data points are represented by the expression ratio and mean±S.D. fold of control in MCF-7 (**a** and **b**) and MDA.MB.231 (**c** and **d**). Each RNA sample represents a pool of RNA samples from two independent biological experiments conducted in triplicate, *P<0.05 *versus* untreated cells.

Because of the ability of proteasome inhibitors to block NF-kB activity, we investigated expression of some established NF-kB target genes, including BAX (BCL2-Associated X Protein), BCL-2 (B-Cell CLL/Lymphoma 2), NFKB2 (Nuclear Factor Of Kappa Light Polypeptide Gene Enhancer In B-Cells 2 (P49/P100)), c-FLIP (CASP8 And FADD-Like Apoptosis Regulator) and IL-8. As shown in Figure 8, BTCI treatment affected gene expression in both breast cancer cell lines, although the effect of BTCI was more pronounced in MDA.MB.231. Interestingly, BTCI-induced IL-8 upregulation in both cell lines, albeit with expression of other NF-kB target genes downregulated and/or unchanged. Although the expression of BAX increased slightly in MCF-7 treated with BTCI in comparison with untreated; however, BTCI treatment did not cause any change in expression of BCL-2, NFKB2 and c-FLIP in MCF-7 cells; while in MDA.MB.231 cells treated with BTCI expression of those genes was markedly downregulated.

## Discussion

Evidence based on animal and epidemiological studies indicates that BBIs, the most extensively studied group of proteasome inhibitors, could act as anti-carcinogenic agents.^13,14,42–45^ The idea of chemopreventive effects of BBIs came from epidemiological studies demonstrating that consumption of soybean products, which are particularly rich in protease inhibitors, is associated with decreased cancer incidence rates.^7,14,42,43^ Although the suppressive effect of BBIs on carcinogenesis has been suggested in a human phase IIa clinical trial,^7^ the underlying mechanisms by which they exert their chemopreventive feature remains unclear.

BTCI is a natural plant protease inhibitor from *V. unguiculata* seeds which belongs to the BBI family. As shown previously^40^ and confirmed in the present study, BTCI exerts inhibitory activity toward the 20S proteasome. The proteasome is phylogenetically ancient and has remained highly conserved throughout eukaryotic evolution, as well as ubiquitously distributed and conserved among distantly related species such as archaea, bacteria and eukaryotes.^46–50^ Given the high-evolutionary conservation of the 20S proteasome, data of its proteolytic activity inhibition can be applicable to those expected in the human 20S proteasome, considering any related eukaryotic source. BTCI was characterized as a potent 20S proteasome inhibitor and may be related to ubiquitin-proteasome pathway inhibition, which was investigated in the present work.

Previously, we showed that BTCI-induced cytotoxic and cytostatic effects on MCF-7 cells associated with DNA fragmentation, lysosome membrane permeabilization and apoptosis.^13^ In the present study, we confirm that BTCI has potent *in vitro* anti-tumor activity due to its ability to induce apoptosis against a panel of breast cancer cells. The ubiquitin-proteasome pathway controls the proteins that are crucial in cellular processes such as cell cycle progression. Recently, it has been reported that under proteasome stress, cell cycle progression was impaired at G2/M phases.^51^ Also, the activation of apoptosis at mitotic arrest depends on the phosphorylation of p38 MAPK.^52,53^ Thus, we suggest that p38/MAPK signaling might be involved in BTCI-induced apoptosis and G2/M arrest in breast cancer cells under proteasome inhibition.

Our findings also demonstrated that BTCI induced a rapid increase in ROS and loss of mitochondrial membrane potential, probably through the proteasome dysfunction; however, the order of these events remain unclear. These observations are consistent with earlier reports showing that inhibition of proteasome activity causes ROS generation.^31,54,55^ In the same line of evidence, it has been reported that proteasome inhibition by MG132 leads to mitochondrial damage and increased ROS production, providing new insights into the mechanism of cell death caused by proteasome dysfunction.^23^

Several lines of evidence suggest a crucial role for ROS as a common and critical denominator in stimulatory and inhibitory cellular signaling events involving NF-*κ*B, which is directly involved in gene regulation associated with various cellular processes such as cell growth, differentiation and apoptosis.^56,57^ Previous studies demonstrated that proteasome inhibition may interact with NF-kB, resulting in cell death in malignant cells.^58–60^ On the other hand, it has been reported that bortezomib, the first proteasome inhibitor used as anticancer drug,^61^ could not inhibit NF-kB p65 nuclear translocation leading to NF-kB activation.^62–64^ We then hypothesized about the possibility that BTCI-induced proteasome impairment may knockdown the transcriptional activity of NF-*κ*B as a possible pathway of apoptotic process. To this end, we investigated expression of some NF-kB-dependent genes. Our findings showed that BTCI treatment inhibited and/or unaltered expression of NF-kB target genes in a cell-type manner; whilst increasing expression of IL-8 in both cell lines.^64,65^ As a consequence of proteasome inhibition, a drop in cellular FLICE-inhibitory protein levels, which is a truncated and inactive form of caspase-8, has been reported in prostate cancer, renal cancer and leukemia.^66,67^ Given this, c-FLIP as a target for cancer therapy seems reasonable. Taken together, our results are in good agreement with previous reports and support the idea that NF-kB may not be a key mechanism of the proteasome inhibitor’s anticancer activity.

Proteasome dysfunction has been shown to induce either the intrinsic or extrinsic pathway for apoptosis, consisting of early release of mitochondrial cytochrome *c* into the cytosol, caspase-9 activation and consequently the activation of caspase-3 and 7; or followed by independent activation of the caspase-8 pathway.^68^ Here, we have observed BTCI-induced apoptosis through the mitochondrial pathway involving caspase-3 activation in MDA-MB-231 cells. Although MCF-7 does not express caspase 3, the increased activity observed in MCF-7 may be due to the method of assessment via DEVD-dependent caspase activity. The assay is based on detection of cleavage of substrate DEVD-AFC that also can detect caspase-7 activity, which is expressed in MCF-7 cells and exhibits a certain degree of functional redundancy with caspase-3. Even though we did not investigate caspase-7 activity, the increase caspase activity observed in MCF-7 might be attributable to the caspase-7 activity. Indeed, mitochondrial membrane permeabilization is a point of no return in many pathways to apoptosis. On the other hand, the caspase-independent cell death resulting from mitochondrial membrane permeabilization does not exhibit the typical hallmarks of apoptosis.^69^ Therefore, it seems plausible to assume that BTCI induces apoptosis in a caspase-dependent manner following mitochondrial damage.

In summary, the present findings showed that BTCI might inhibit the cancer cell function directly by blocking the 20S proteasome core cavity, and indirectly by the generation of molecular intermediates responsible for oxidative stress. As cancer cells are generally more sensitive than normal cells to the inhibition of proteasome activity, proteasome inhibitors are being employed as chemopreventive agents. BTCI is safe, of low cost and can be taken as a part of a daily diet. Thus, the potential of BTCI for combinational therapies for breast cancer is promising.

## Materials and methods

### Materials

MDA.MB.231 (highly invasive human breast cells), MCF-7 (human breast adenocarcinoma cells) and MCF-10A (normal mammary epithelial cells) were purchased from Rio de Janeiro Cell Bank (RJCB). Caspase 3 Assay Kit and annexin V-FITC Apoptosis Detection Kit were obtained from Abcam Inc. (Cambridge, MA, USA). 3-(4,5-dimethylthiazol-2-yl)-2,5-diphenyltetrazolium bromide (MTT), PI, Fluorometric Intracellular ROS Kit, Mitochondrial Membrane Potential Kit and all cell culture reagents were purchased from Sigma (St Louis, MO, USA).

### Cell culture

Human breast cancer cell lines MCF-7 and MDA.MB.231 were maintained in Eagle’s minimum essential medium and Leibovitz’s L-15 medium, respectively. The media were supplemented with 10–20% (v/v) of fetal bovine serum and antibiotics (100 U/ml penicillin and 100 *μ*g/ml streptomycin). MCF-10A cells were maintained in Mammary Epithelial Cell Growth Medium supplemented with 5% horse serum, 20 ng/ml epidermal growth factor, 10 lg/ml insulin, 0.5 lg/ml hydrocortisone, 100 ng/ml cholera toxin and antibiotics (100 IU/ml penicillin and 100 lg/ml streptomycin). Cells were maintained at 37 °C under humidified air containing 5% CO_2_ for MCF-7 and MCF-10A and without CO_2_ in the case of MDA.MB.231.

### BTCI and proteasome 20S purification

BTCI was purified as previously described.^39,41^ In brief, *V. unguiculata* seeds were triturated, homogenized in water and submitted to 2.5% (v/v) trichloroacetic acid and 70% (w/v) ammonium sulfate precipitation. The obtained CE was dialyzed, lyophilized and stored at −20 ^o^C. BTCI was purified from CE by ion exchange chromatography using DEAE-cellulose column (Sigma). The purity and molecular mass of BTCI were evaluated by MALDI-TOF/MS using an UltraFlex III (Bruker Daltonics, Bremen, Germany). BTCI concentration was determined using its specific absorption coefficient $A_{280\mathrm{nm}}^{1\%}=8.23$ and molecular mass of 9.1 kDa. Mammal 20S proteasome was purified from horse erythrocytes and used to analyze cytotoxic effects of BTCI on human breast adenocarcinoma cells, given its phylogenetic conservation in the biogenesis,^70,71^ as well as in the tridimensional structure in all eukaryotes organisms.^47^ Moreover, these cells were chosen based on available facilities and the well-established purification method for the 20S proteasome in large amounts.^72^ In brief, horse erythrocytes lysates were obtained in 1 mM dithiothreitol (DTT) after removal of membranes. Cell lysates were applied onto sequential ion exchange DEAE-Sepharose and mono-Q columns, using an FPLC facility (GE Healthcare, Pharmacia, Bio-Rad Laboratories, Inc., Foster City, CA, USA) and sucrose density gradient ultracentrifugation.^72^

### Effect of BTCI on enzymatic activity of the 20S proteasome

Fluorogenic substrates *t-butyloxycarbonyl*-*L*-*leucyl*-*L*-*arginyl*-L-*arginine-4*-*methylcoumaryl*-*7-amide* (Boc-Leu-ArgArg-AMC), *benzyloxycarbonyl-L-leucyl-L-leucyl-L-glutamyl- naphthylamide* (Z-Leu-Leu-Glu-*β*NA) and *N-succinyl- Leu-Leu-Val-Tyr-AMC* (Suc-Leu-Leu-Val-Tyr) were used for trypsin, caspase and chymotrypsin-like activities, respectively as previously described.^40^ In brief, purified 20S proteasome (2.0 *μ*g/ml) was incubated with BTCI (2.0 to 30.0 *μ*g/ml) and 62.5 *μ*g/ml of the fluorogenic substrates in 20.0 mM Tris-HCl pH 7.5, 1.0 mM EDTA, 1.0 mM NaN3, 1.0 mM DTT. The assays were carried out in triplicate, at room temperature for 60 min. The hydrolysis of fluorogenic substrates was monitored at 480 nm for chymotrypsin-like and trypsin-like activities, and 410 nm for caspase-like activities.^40^ The inhibition constant, K_I_ was calculated by nonlinear regression using the GRAFIT program version 3 (Erithacus Software Ltd., Middlesex, UK). The control for this experiment was established using the proteasome inhibitor MG132 (*carbobenzoxylleucyl-leucyl-leucinaI-H*) at a similar concentration to that used when BTCI displayed 80–95% of protease inhibition.

### MTT assay

Cells were digested with trypsin, harvested, adjusted to a density of 2×10^4^ cells/ml and seeded to 96-well plates at volume 200*μ*l per well. After 24 h when cells formed a monolayer, BTCI was added to the medium at different concentrations. After incubation for 24 h, MTT (3-(4, 5-dimethylthiazol-2-yl)-2, 5-diphenyltetrazolium Bromide; 5 mg/ml) was added (10 *μ*l per well). Cells were then incubated at 37 °C for a further 3 h, culture supernatant removed and dimethyl sulfoxide added (100 *μ*l per well). Cells were incubated on a shaker at 37 °C for 10 min until crystals were completely dissolved. The absorbance at 570 nm was determined using a SpectraMax M3 microplate reader (Molecular Devices, Sunnyvale, CA, USA), then correlated with cell viability. Each experiment was conducted in quintuplicate at least twice.

### Annexin V-FITC and PI staining

Subsequent to treatment, cells were harvested and washed twice with phosphate-buffered saline (PBS). A total of 5 *μl* annexin V-FITC and 5 *μ*l PI were then added according to the manufacturer’s instructions. Cells were then incubated at room temperature, in the dark, for 15 min and analyzed using a flow cytometer system (FACScan; BD Biosciences, San Jose, CA, USA).

### DNA flow cytometric analysis

After treatment with BTCI, cells were digested with trypsin, harvested, washed twice with ice-cold PBS, fixed with 75% ethanol at −20 °C overnight, washed again and then incubated with RNase A (25 *μ*g/ml, Bio Basic Inc., Markham, Ontario, Canada) at 37 °C for 30 min. Cells were washed once with PBS and incubated with PI (50 *μ*g/ml) for 30 min at room temperature in the dark. The cells were re-suspended in 500 *μl* PBS and subjected to flow cytometry on a Cytomics FC500 flow cytometer (FACScan; BD Biosciences). A sub G0/G1 population seen to the left of the G0/G1 peak represents DNA fragmentation caused by apoptosis.

### JC-10 mitochondrial membrane potential assay

Mitochondrial membrane potential was measured using JC-10 Molecular Probes staining from Sigma (MAK159) as per manufacturer´s instructions. In brief, cells were cultured on 96-well white-walled clear-bottom plates and treated with BTCI for 24 h. Then, 50 *μ*l of JC-10 dye-loading solution was added to each well and incubated for 30 min. The samples were read on a SpectraMax M3 microplate reader (Molecular Devices), measuring fluorescence intensities (Ex/Em= 485/515 nm and Ex/Em= 540/590 nm). The change of mitochondrial membrane potential was measured as the ratio between aggregate (Em=515 nm) and monomeric forms (Em= 590 nm) of JC-10. Increasing ratios indicate mitochondrial membrane depolarization.

### Caspase-3 activity assay

Caspase-3 activity was measured using the Caspase-3 assay kit from Abcam Inc. (ab39383) as per manufacturer´s instructions. In brief, samples were trypsinized and re-suspended in cell lysis buffer and allowed to lyse for 10 min. Reaction buffer containing 5 *μ*l (DTT) and the 10 *μ*l (DEVD-AFC) substrate was added and the mixture was incubated at 37 °C for 2 h. The samples were read on a SpectraMax M3 microplate reader (Molecular Devices), using a 400 nm excitation and 505 nm emission.

### Determination of intracellular generation of ROS

The production of intracellular ROS was estimated fluorimetrically using the oxidation sensitive fluorescent kit from Sigma (MAK144) that provides a sensitive, one-step fluorometric assay to detect intracellular ROS (especially superoxide and hydroxyl radicals) in live cells. In brief, cells were treated with BTCI for 30, 60, 90, 120 and 180 min. Then, 100 *μ*l of Master Reaction Mix was added and samples were incubated at 37 °C for 60 min. The samples were read on a SpectraMax M3 microplate reader (Molecular Devices), using a 540 nm excitation and 570 nm emission.

### Real-time polymerase chain reaction

The amount of BAX (BCL2-Associated X Protein), BCL-2(B-Cell CLL/Lymphoma 2), NFKB2 (Nuclear Factor of Kappa Light Polypeptide Gene Enhancer in B-Cells 2 (P49/P100)), CFLAR (CASP8 and FADD-Like Apoptosis Regulator) and IL-8 mRNA was assessed by real-time reverse transcription-PCR (RT-PCR) using a 7500 Fast Real-Time PCR System (Applied Biosystems, Foster City, CA, USA). Total RNA was isolated using Trizol reagent (Invitrogen, Carlsbad, CA, USA) according to the manufacturer´s protocol. RNA (1 *μ*g) was used for cDNA synthesis using QuantiTect Reverse Transcription Kit (QIAGEN, Valencia, CA, USA). Real-time PCR was carried out using the Fast SYBR as a detection method. Primer sequences, as presented in Table 1, were purchased from Exxtend (Rio de Janeiro, Brazil). In addition, the amount of *β*-actin mRNA was quantified in all samples as an internal constitutive control. The concentration of gene-specific mRNA in treated samples relative to untreated samples was calculated using the 2 –^ΔΔCT^ formula as follows: 2^–ΔΔCT^=[(C_T_ gene of interest—C_T_ internal control) treatment group]—[(C_T_ gene of interest—C_T_ internal control) control group]. Results are shown as fold changes compared with the control.^73,74^ All experiments were conducted in triplicate.

### Statistical analysis

All data are presented as the mean±S.D. of at least two independent experiments conducted in triplicate. Statistical analysis was performed using one-way ANOVA followed by Dunnett’s multiple comparison test using SPSS 19.0 software (SPSS Inc., Chicago, IL, USA). Differences were considered to be significant at a value of *P*<0.05.

## Figures and Tables

**Figure 6:**
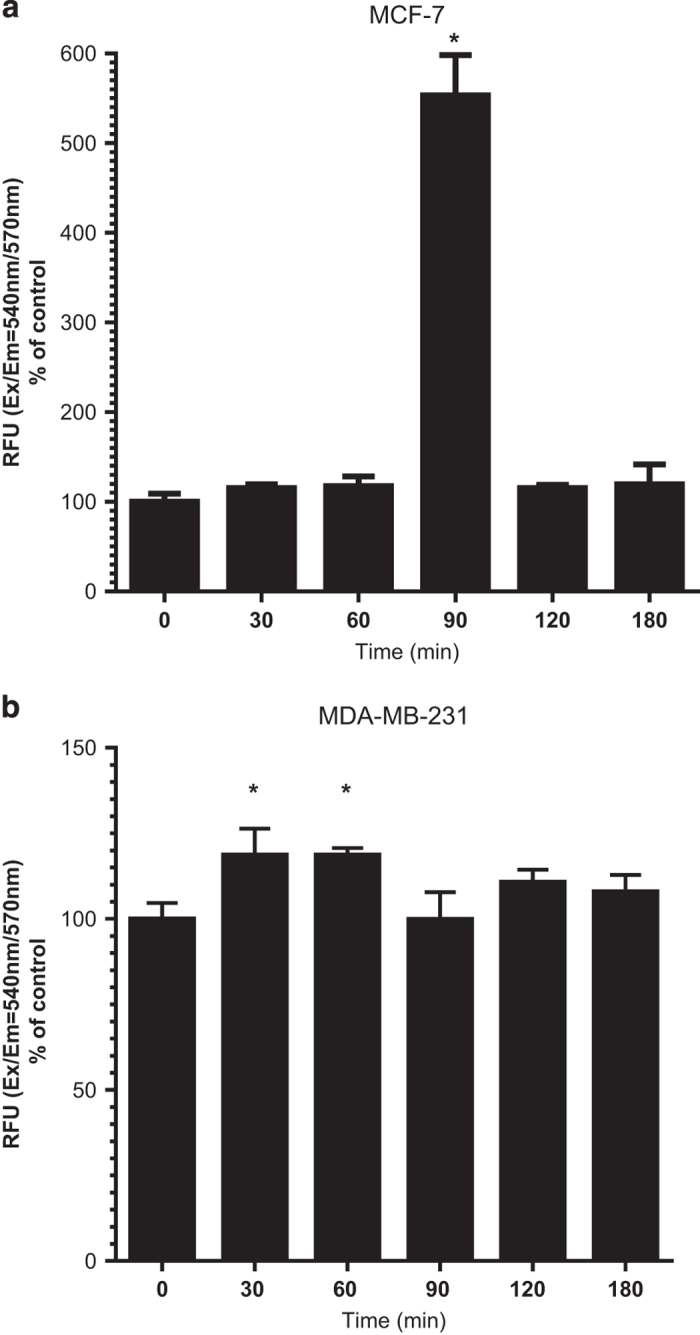
BTCI enhanced the generation of ROS in human breast adenocarcinoma cells. (**a**) MCF-7 and (**b**) MDA-MB-231 cells were incubated with BTCI at the concentration of 150 *μ*M and 100 *μ*M, respectively, for 30, 60, 90 and 180 min. The production of intracellular ROS was estimated fluorimetrically using Fluorometric Intracellular Ros Kit. Results are presented as mean±S.D. of two separate experiments conducted in triplicate, *P<0.001 *versus* untreated cells.

**Table 1:** Human gene-targeting primers used in this study

*Primer*	*Sequence (5'—3')*
IL-8 forward	ACTGAGAGTGATTGAGAGTGGAC
IL-8 reverse	AACCCTCTGCACCCAGTTTTC
NFKB2 forward	ATGGAGAGTTGCTACAACCCA
NFKB2 reverse	CTGTTCCACGATCACCAGGTA
CFLAR forward	AGAGTGAGGCGATTTGACCTG
CFLAR reverse	GTCCGAAACAAGGTGAGGGTT
BAX forward	CCCGAGAGGTCTTTTTCCGAG
BAX reverse	CCAGCCCATGATGGTTCTGAT
BCL-2 forward	TACAGGCTGGCTCAGGACTAT
BCL-2 reverse	CGCAACATTTTGTAGCACTCTG
ACTB forward	GGATGCAGAAGGAGATCACTG
ACTB reverse	CGATCCACACGGAGTACTTG
