# Correction to: A Bowman–Birk inhibitor induces apoptosis in human breast adenocarcinoma through mitochondrial impairment and oxidative damage following proteasome 20S inhibition

**DOI:** 10.1038/s41420-024-02155-4

**Published:** 2024-09-18

**Authors:** A Mehdad, G Xavier Reis, AA Souza, JARG Barbosa, MM Ventura, SM de Freitas

**Affiliations:** 1 Laboratory of Molecular Biophysics, Institute of Biological Sciences, University of Brasilia, Brasilia, Brazil; 2 Faculty of Medicine, Postgraduate program in Molecular Pathology, University of Brasilia, Brasilia, Brazil

Correction to: *Cell Death Discovery* 10.1038/cddiscovery.2015.67, published online 21 March 2016

In this article the author’s name G Brumana is given erroneously. It should read: G Xavier Reis.

The original article has been corrected.

